# Supplementary material for: Improving Mechanical Properties for Extrusion-Based Additive Manufacturing of Poly(Lactic Acid) by Annealing and Blending with Poly(3-Hydroxybutyrate)
Source: Polymers (Basel). 2019 Sep 19;11(9):1529. doi: 10.3390/polym11091529 (PMC6780387; doi:10.3390/polym11091529)
Supplement: Supplementary file 1 [file polymers-11-01529-s001.pdf]

polymers

Supplementary Materials

Improving Mechanical Properties for Extrusion-Based Additive Manufacturing of Poly(lactic acid) by Annealing and Blending with Poly(3-hydroybutyrate)

Sisi Wang [1], Lode Daelemans [2], Rudinei Fiorio [1], Maling Gou [3], Dagmar R. D'hooge [2,4], Karen De Clerck [2,*], Ludwig Cardon [1,*]

1. Extra Information for Materials

MFI and TGA data for the materials in this study are shown in Table S1. The melt flow rate of PLA, PHB and PLA/PHB filaments ranged from 180 °C to 230 °C with load of 2.16 kg. The degradation behavior of the filaments was measured by thermogravimetrical analysis (TGA) under N2 flow. The samples were heated from room temperature to 550 °C at a rate of 10 °C min^{-1}.

Table S1. MFI and TGA data for PHB, PLA and PLB/PHB filaments.

Material		PHB	PLA	PLA/PHB
MFI (g) (T, 2.16 kg)	T=180 °C	8.2 ± 0.5	2.0 ± 0.2	5.3 ± 0.3
	T=190 °C	24.1 ± 0.5	4.3 ± 0.2	7.5 ± 0.3
	T=210 °C	ca. 57.6	8.9 ± 0.2	16.9 ± 1.4
	T=230 °C		17.9 ± 0.8	
DSC data	Tm (°C)	172	176	175
TGA data	T at mass loss of 5% (°C)	242.4	334.9	279.3
	Maximum loss peak (°C)	286.0	373.8	291.0, 346.4

The MFI value for PHB and the PHB/PLA blends was much larger than that of PLA alone and the onset degradation temperature ($T_{5\% \text{ lost}}$) of PHB was much lower than that of PLA. Notably, PHB is more sensitive to temperature than PLA. Based on previous studies, PHB/PLA can be printed using a lower temperature than PLA, and the material performs well when the melt flow index is close to 10 g (10 min)$^{-1}$ [1]. PLA and PLA/PHB showed the best mechanical properties at 210 °C and 190 °C, respectively (Table S2). The impact strength was lower and was independent of temperature due to insufficient flow at low temperature or thermal degradation at high temperature. These results indicate that pure PLA and the PLA/PHB blend should be printed at 210 °C and 190 °C, respectively.

Table S2. Basic mechanical properties of printed PLA and PLA/PHB.

Sample	T_{Nozzle} (°C)	Notched Impact Strength (kJ m^{-2})	Modulus (MPa)	Tensile Stress at Maximum Load (MPa)	Tensile Strain at Break (%)
PLA	190	3.7 ± 0.3	3554 ± 213	61.1 ± 2.4	3.3 ± 0.3
	210	4.1 ± 0.5	3520 ± 158	66.2 ± 2.3	3.8 ± 0.4
	230	3.7 ± 0.3	3455 ± 105	61.2 ± 1.2	4.1 ± 0.5
PLA/PHB	180	4.5 ± 0.1	3104 ± 136	48.9 ± 0.8	7.4 ± 0.8
	190	4.8 ± 0.5	3382 ± 114	55.4 ± 0.9	6.8 ± 0.4
	210	4.3 ± 0.5	3281 ± 213	52.0 ± 2.4	6.8 ± 0.7

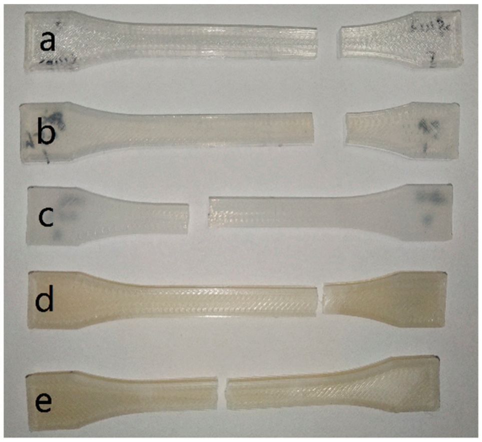

Figure S1. Optical image of representative printed bars (**a**) PLA, (**b**) PLA-80°C 0.5h, (**c**) PLA-100°C 0.5h, (**d**) PLA/PHB, (**e**) PLA/PHB-80°C 0.5h.

Both PLA and PLA/PHB were well printed and won't wrap after annealing. The non-annealed PLA was transparent, after annealing it turned opaque. PLA/PHB blend showed the same appearance before and after annealing.

2. Extra Information on Results

2.1. DSC Results

PLA/PHB blend presents lower T_g, T_{cc} and smaller ΔH_{cc} than pure PLA, proving that PHB enhanced the crystallization ability of PLA. The annealed samples all reach maximum crystallinity with no recrystallization peak shown after annealing.

Table S3. Thermal data for PLA and PLA/PHB samples before and after annealing; related to Figure 1a; errors are < 2%.

Sample	T_g (°C)	T_{cc} (°C)	ΔH_{cc} (J g^{-1})	T_m (°C)	ΔH_m (J g^{-1})	ΔH_m+ΔH_{cc} (J g^{-1})	x_c (%)
PLA	58	98	-33	176	51	18	20
PLA-80°C0.5h				176	53	53	57
PLA-80°C1h				176	52	52	56
PLA-80°C2h				175	53	54	58
PLA-100°C0.5h				177	51	51	55
PLA-100°C1h				177	51	51	54
PLA-100°C2h				176	50	50	53
PHB				172	70	70	48
PLA/PHB	52	85	-18	176	55	38	
PLA/PHB-80°C0.5h				173	56	56	
PLA/PHB-80°C1h				176	55	55	
PLA/PHB-80°C2h				175	56	57	
PLA/PHB-100°C0.5h				174	56	56	
PLA/PHB-100°C1h				174	54	54	
PLA/PHB-100°C2h				174	54	54	

2.2. XRD Result

As reported in the literature, the XRD for PHB shows two strong scattering intensity peaks at a 2θ value of ca. 13° and 17°, which can be assigned to the (020) and (110) plane of the orthorhombic unit cell [2–6]. Here, weak crystalline peaks were obtained at 10°, 20°, 22°, 25°, and 27°, respectively,

corresponding to planes of (110), (021), (121), (040), and (200) (Figure 3b) [3,5]. Some of these PHB peaks coincided with the PLA diffraction peaks [2,6–9]. The XRD pattern of neat PHB is not shown due to difficulties in obtaining a flat printing sample. Non-annealed PLA/PHB showed reflection peaks at 10.3°, 14.4°, and a diffuse peak around 17°, implying that PHB, but not PLA, crystallized completely during the printing process (Figure 3b). Annealed PLA/PHB displayed a strong reflection at 10.3° (110_{PHB}), 17.5° ($110/200_{PLA}$) and 19.8° (203_{PLA}), and weaker reflections at 14.4° and 29.6°, reflecting higher crystallinity than the non-annealed sample, which is in accordance with the DSC results.

The 110/200 and 203 planes of PLA/PHB were shifted upward compared to PLA, suggesting that PHB increases the space for PLA between the crystal planes. However, the intensity of the 110/200 and 203 peaks for PLA/PHB was decreased slightly and broadened compared to that for PLA. This difference can be attributed to the PLA/PHB blends interfering with the spherulite structure of each other due to the different crystallization kinetics [10].

2.3. Mechanical Property of the Samples

Table S4. Tensile property of PLA and PLA/PHB blend before and after annealing.

Sample	No.	Modulus (MPa)	Tensile Stress at Yield (MPa)	Tensile Stress at Maximum Load (MPa)	Tensile Strain at Break (%)	Notched Impact Strength (kJ m^{-2})
PLA	1-1	3520 ± 158	55.7 ± 2.4	66.2 ± 2.3	3.8 ± 0.4	4.1 ± 0.5
PLA-80°C0.5h	1-2	3381 ± 41	57.2 ± 2.2	68.8 ± 2.4	3.2 ± 0.4	7.9 ± 2.1
PLA-80°C1h	1-3	3540 ± 100	58.8 ± 1.0	70.0 ± 1.6	4.3 ± 1.9	9.7 ± 1.3
PLA-80°C2h	1-4	3627 ± 120	57.3 ± 4.1	69.6 ± 1.4	3.3 ± 0.6	13.4 ± 2.2
PLA-100°C0.5h	1-5	3940 ± 332	59.5 ± 3.8	67.6 ± 1.5	2.2 ± 0.2	12.5 ± 1.5
PLA-100°C1h	1-6	3801 ± 268	62.1 ± 3.0	69.4 ± 1.9	2.7 ± 0.6	13.2 ± 0.7
PLA-100°C2h	1-7	3752 ± 134	60.1 ± 0.6	67.4 ± 0.9	2.7 ± 0.3	15.6 ± 0.6
PLA/PHB	2-1	3382 ± 114	49.4 ± 2.0	55.4 ± 0.9	6.8 ± 0.4	4.8 ± 0.5
PLA/PHB-80°C0.5h	2-2	3829 ± 267	45.6 ± 2.2	57.8 ± 1.2	4.2 ± 0.2	5.8 ± 1.7
PLA/PHB-80°C1h	2-3	3757 ± 95	46.5 ± 2.3	57.7 ± 1.6	3.8 ± 0.9	6.4 ± 0.4
PLA/PHB-80°C2h	2-4	3762 ± 94	44.1 ± 2.0	55.5 ± 1.1	3.4 ± 0.5	6.4 ± 0.1
PLA/PHB-100°C0.5h	2-5	3948 ± 151	46.0 ± 1.8	58.3 ± 0.8	3.0 ± 0.7	5.6 ± 0.3
PLA/PHB-100°C1h	2-6	3875 ± 243	46.1 ± 3.8	56.3 ± 1.9	3.4 ± 1.0	6.0 ± 0.1
PLA/PHB-100°C2h	2-7	4005 ± 115	45.9 ± 1.5	57.9 ± 1.8	3.7 ± 1.1	6.5 ± 0.4

Tensile and impact results of PLA and PLA/PHB blend before and after annealing are shown in Table S4. Analysis of variance (ANOVA) was performed on the dataset for tensile tests using SPSS software to check data reliability (Table S5). Sig. values <0.05 indicated significant differences between the two groups.

Table S5. One-way ANOVA results for tensile properties: Modulus, Tensile stress at Maximum Load, stress at break. Sig. value < 0.05 means there is a difference between the two samples.

		Sig. Value					Sig. Value		
Comparation between		Modulus	Tensile Stress at Maximum Load	Tensile Strain at Break	Comparation between		Modulus	Tensile Stress at Maximum Load	Tensile Strain at Break
1-1	1-2	>0.05	>0.05	>0.05	2-1	2-2	<0.05	>0.05	<0.05
	1-3	>0.05	<0.05	>0.05		2-3	<0.05	>0.05	<0.05
	1-4	>0.05	>0.05	>0.05		2-4	<0.05	>0.05	<0.05
	1-5	<0.05	>0.05	>0.05		2-5	<0.05	<0.05	<0.05
	1-6	>0.05	>0.05	>0.05		2-6	<0.05	>0.05	<0.05
	1-7	>0.05	>0.05	>0.05		2-7	<0.05	>0.05	<0.05
1-2	1-1	>0.05	>0.05	>0.05	2-2	2-1	<0.05	>0.05	<0.05
	1-3	>0.05	>0.05	>0.05		2-3	>0.05	>0.05	>0.05
	1-4	>0.05	>0.05	>0.05		2-4	>0.05	>0.05	>0.05
	1-5	<0.05	>0.05	>0.05		2-5	>0.05	>0.05	>0.05
	1-6	<0.05	>0.05	>0.05		2-6	>0.05	>0.05	>0.05
	1-7	>0.05	>0.05	>0.05		2-7	>0.05	>0.05	>0.05
1-3	1-1	>0.05	<0.05	>0.05	2-3	2-1	<0.05	>0.05	<0.05
	1-2	>0.05	>0.05	>0.05		2-2	>0.05	>0.05	>0.05
	1-4	>0.05	>0.05	>0.05		2-4	>0.05	>0.05	>0.05
	1-5	<0.05	>0.05	<0.05		2-5	>0.05	>0.05	>0.05
	1-6	>0.05	>0.05	>0.05		2-6	>0.05	>0.05	>0.05
	1-7	>0.05	>0.05	>0.05		2-7	>0.05	>0.05	>0.05
1-4	1-1	>0.05	>0.05	>0.05	2-4	2-1	<0.05	>0.05	<0.05
	1-2	>0.05	>0.05	>0.05		2-2	>0.05	>0.05	>0.05
	1-3	>0.05	>0.05	>0.05		2-3	>0.05	>0.05	>0.05
	1-5	>0.05	>0.05	>0.05		2-5	>0.05	>0.05	>0.05
	1-6	>0.05	>0.05	>0.05		2-6	>0.05	>0.05	>0.05
	1-7	>0.05	>0.05	>0.05		2-7	>0.05	>0.05	>0.05
1-5	1-1	<0.05	>0.05	>0.05	2-5	2-1	<0.05	<0.05	<0.05
	1-2	<0.05	>0.05	>0.05		2-2	>0.05	>0.05	>0.05
	1-3	<0.05	>0.05	<0.05		2-3	>0.05	>0.05	>0.05
	1-4	>0.05	>0.05	>0.05		2-4	>0.05	>0.05	>0.05
	1-6	>0.05	>0.05	>0.05		2-6	>0.05	>0.05	>0.05
	1-7	>0.05	>0.05	>0.05		2-7	>0.05	>0.05	>0.05
1-6	1-1	>0.05	>0.05	>0.05	2-6	2-1	<0.05	>0.05	<0.05
	1-2	<0.05	>0.05	>0.05		2-2	>0.05	>0.05	>0.05
	1-3	>0.05	>0.05	>0.05		2-3	>0.05	>0.05	>0.05
	1-4	>0.05	>0.05	>0.05		2-4	>0.05	>0.05	>0.05
	1-5	>0.05	>0.05	>0.05		2-5	>0.05	>0.05	>0.05
	1-7	>0.05	>0.05	>0.05		2-7	>0.05	>0.05	>0.05
1-7	1-1	>0.05	>0.05	>0.05	2-7	2-1	<0.05	>0.05	<0.05
	1-2	>0.05	>0.05	>0.05		2-2	>0.05	>0.05	>0.05
	1-3	>0.05	>0.05	>0.05		2-3	>0.05	>0.05	>0.05
	1-4	>0.05	>0.05	>0.05		2-4	>0.05	>0.05	>0.05
	1-5	>0.05	>0.05	>0.05		2-5	>0.05	>0.05	>0.05
	1-6	>0.05	>0.05	>0.05		2-6	>0.05	>0.05	>0.05

2.4. VST of Samples

Table S6. Vicat softening temperature of samples.

Sample	VST (°C)	Sample	VST (°C)
PLA	60.3	PLA/PHB	153.4
PLA-80°C0.5h	164.8	PLA/PHB-80°C0.5h	157.8
PLA-80°C1h	162.8	PLA/PHB-80°C1h	157.7
PLA-80°C2h	163.2	PLA/PHB-80°C2h	157.7
PLA-100°C0.5h	164.7	PLA/PHB-100°C0.5h	158.4
PLA-100°C1h	164.7	PLA/PHB-100°C1h	158.5
PLA-100°C2h	163.6	PLA/PHB-100°C2h	158.2

2.5. Dimension Stability of Samples

Density test: A Precisa XR 2055M-DR was used for density determinations based on the Archimedes principle. As a reference, 99.8% ethanol with a density of 0.803 g cm^{-3} was used. Calibration was based on a standardized glass rod. The density was measured at room temperature (20 °C) according to ISO 1183.

Dimension changes: Length, width and thickness of the printed bars were measured manually before and after annealing using a caliper to determine the dimension stability after annealing. Negative values in Table S8 represent the percentage decrease in dimension, whereas positive values

represent the percentage increase in the dimension after annealing compared to that before annealing.

Density results and dimension changes are shown in Tables S7 and S8, respectively. The percentage difference in density of annealed samples is within 1% of the non-annealed sample and hardly any voids were seen between the printed strands. The dimensions of non-annealed and annealed samples varied by less than 3%. Meanwhile, the thickness increased but the width and length of the samples decreased after annealing, in accordance with previous findings [11]. The increase in thickness may be due to the inner stress of layers during printing, wherein strands are forced onto earlier layers that then stretch back to a rounder shape after annealing. Given the small variations seen among density and dimensions, dimension changes that occur during printing should have virtually no effect on the mechanical properties of the samples.

Table 7. Density of bars before and after annealing.

Sample	Average Value (g cm^{-3})	Difference in Density Compare to Non-Annealed Sample (%)	Sample	Average Value (g cm^{-3})	Difference in Density Compare to Non-Annealed Sample (%)
PLA pellet	1.263 ± 0.002		PHB pellet	1.226 ± 0.006	
PLA filament	1.215 ± 0.008		PHB filament	1.242 ± 0.001	
			PLA/PHB filament	1.224 ± 0.008	
PLA	1.236 ± 0.009	/	PLA/PHB	1.245 ± 0.001	/
PLA-80°C0.5h	1.238 ± 0.007	0.16	PLA/PHB-80°C0.5h	1.248 ± 0.001	0.24
PLA-80°C1h	1.246 ± 0	0.81	PLA/PHB-80°C1h	1.247 ± 0.002	0.12
PLA-80°C2h	1.246 ± 0	0.81	PLA/PHB-80°C2h	1.246 ± 0.002	0.04
PLA-100°C0.5h	1.249 ± 0.001	1.05	PLA/PHB-100°C0.5h	1.245 ± 0.003	0.02
PLA-100°C1h	1.242 ± 0.003	0.51	PLA/PHB-100°C1h	1.247 ± 0.001	0.16
PLA-100°C2h	1.242 ± 0.002	0.45	PLA/PHB-100°C2h	1.249 ± 0.003	0.29

Table 8. Change in bar dimensions before and after annealing.

	Thickness (%)	Width (%)	Length (%)		Thickness (%)	Width (%)	Length (%)
PLA-80°C0.5h	2.49	-0.68	-1.24	PLA/PHB-80°C0.5h	2.51	-0.53	-0.6
PLA-80°C1h	1.86	-0.60	-1.34	PLA/PHB-80°C1h	1.99	-0.8	-0.54
PLA-80°C2h	1.68	-0.73	-1.54	PLA/PHB-80°C2h	1.74	-0.7	-0.56
PLA-100°C0.5h	3.05	-0.90	-1.42	PLA/PHB-100°C0.5h	0.75	-0.59	-0.78
PLA-100°C1h	3.18	-1.05	-1.41	PLA/PHB-100°C1h	1.26	-0.6	-0.65
PLA-100°C2h	3.39	-1.15	-1.41	PLA/PHB-100°C2h	2.27	-0.79	-0.92

References

1. Wang, S.; Capoen, L.; D'hooge, D. R.; Cardon, L. Can the melt flow index be used to predict the success of fused deposition modelling of commercial poly(lactic acid) filaments into 3D printed materials? *Plast., Rubber Compos.* **2017**, *47*, 1, 9–16, doi: 10.1080/14658011.2017.1397308.
2. Zhijiang, C.; Guang, Y. Optical nanocomposites prepared by incorporating bacterial cellulose nanofibrils into poly (3-hydroxybutyrate). *Mater. Lett.* **2011**, *65*, 2, 182–184.
3. Wei, L.; McDonald, A. G.; Stark, N. M. Grafting of bacterial polyhydroxybutyrate (PHB) onto cellulose via in situ reactive extrusion with dicumyl peroxide. *Biomacromolecules* **2015**, *16*, 3, 1040–9, doi: 10.1021/acs.biomac.5b00049.
4. Arrieta, M. P.; López, J.; López, D.; Kenny, J. M.; Peponi, L. Development of flexible materials based on plasticized electrospun PLA–PHB blends: Structural, thermal, mechanical and disintegration properties. *Eur. Polym. J.* **2015**, *73*, 433–446, doi: 10.1016/j.eurpolymj.2015.10.036.
5. D'Amico, D. A.; Iglesias Montes, M. L.; Manfredi, L. B.; Cyras, V. P. Fully bio-based and biodegradable polylactic acid/poly(3-hydroxybutirate) blends: Use of a common plasticizer as performance improvement strategy. *Polym. Test.* **2016**, *49*, 22–28, doi: 10.1016/j.polymertesting.2015.11.004.
6. Arrieta, M. P.; Fortunati, E.; Dominici, F.; Rayon, E.; Lopez, J.; Kenny, J. M. Multifunctional PLA-PHB/cellulose nanocrystal films: processing, structural and thermal properties. *Carbohydr. Polym.* **2014**, *107*, 16–24, doi: 10.1016/j.carbpol.2014.02.044.

7. Nagarajan, V.; Zhang, K.; Misra, M.; Mohanty, A. K. Overcoming the Fundamental Challenges in Improving the Impact Strength and Crystallinity of PLA Biocomposites: Influence of Nucleating Agent and Mold Temperature. *ACS Appl. Mater. Interfaces* **2015**, *7*, 21, 11203–14, doi: 10.1021/acsami.5b01145.
8. Nam, J. Y.; Ray, S. S.; Okamoto, M. Crystallization Behavior and Morphology of Biodegradable Polylactide/Layered Silicate Nanocomposite. *Macromolecules* **2003**, *36*, 7126–7131.
9. Wang, L.; Gramlich, W. M.; Gardner, D. J. Improving the impact strength of Poly(lactic acid) (PLA) in fused layer modeling (FLM). *Polymer* **2017**, *114*, 242–248, doi: 10.1016/j.polymer.2017.03.011.
10. Ye, L.; Ye, C.; Xie, K.; Shi, X.; You, J.; Li, Y. Morphologies and Crystallization Behaviors in Melt-Miscible Crystalline/Crystalline Blends with Close Melting Temperatures but Different Crystallization Kinetics. *Macromolecules* **2015**, *48*, 23, 8515–8525, doi: 10.1021/acs.macromol.5b01904.
11. Rangisetty, S.; Peel, L. D. In The Effect of Infill Patterns and Annealing on Mechanical Properties of Additively Manufactured Thermoplastic Composites, ASME 2017 Conference on Smart Materials, Adaptive Structures and Intelligent Systems, American Society of Mechanical Engineers: **2017**; pp V001T08A017-V001T08A017.

© 2019 by the authors. Submitted for possible open access publication under the terms and conditions of the Creative Commons Attribution (CC BY) license (http://creativecommons.org/licenses/by/4.0/).
